# EGFR-TKI对肺癌新生淋巴管的影响及其意义

**DOI:** 10.3779/j.issn.1009-3419.2014.12.02

**Published:** 2014-12-20

**Authors:** 明辉 蔡, 新颖 杨, 宝红 姜, 福康 滕, 雁 潘, 锋 毛, 阳 申屠

**Affiliations:** 1 200030 上海，上海交通大学附属胸科医院/上海市肺部肿瘤临床医学中心胸外科 Department of Thoracic Cancer, Shanghai Chest Hospital, Shanghai Jiaotong University, Shanghai Lung Tumor Clinical Medical Center, Shanghai 200030, China; 2 201203 上海，中国科学院上海药物研究所 Shanghai Institute of Materia Medical, Chinese Academy of Sciences, Shanghai 201203, China; 3 200030 上海，上海交通大学附属胸科医院药剂科 Department of Pharmacy, Shanghai Chest Hospital, Shanghai Jiaotong University, Shanghai 200030, China

**Keywords:** 肺肿瘤, *EGFR*突变, EGFR-TKI, 新生淋巴管, Lung neoplasms, *EGFR* mutation, EGFR-TKI, Lymphangiogenesis

## Abstract

**背景与目的:**

探索表皮生长因子受体酪氨酸激酶抑制剂（epidermal growth factor receptor-tyrosine kinase inhibitor, EGFR-TKI）对*EGFR*突变肺癌新生淋巴管的影响，探讨靶点治疗对新生淋巴管的抑制作用及其在肺癌治疗中所发挥的作用。

**方法:**

采用EGFR双位点突变的NCI-H1975肺癌细胞株构建小鼠移植瘤模型。设立溶剂对照组和EGFR-TKI给药组，每组5只小鼠，观察EGFR-TKI对小鼠移植瘤的生长抑制作用；运用淋巴管内皮特异性抗体D2-40，采用免疫组织化学的方法，观察新生淋巴管的密度、面积、最大径，探讨EGFR-TKI对于肺癌组织淋巴管新生的影响。

**结果:**

EGFR-TKI给药组小鼠肿瘤重量、肿瘤相对体积小于溶剂对照组。EGFR-TKI给药组小鼠平均新生淋巴管密度为6.44个/例，溶剂对照组小鼠平均新生淋巴管密度为10.70个/例，EGFR-TKI给药组小鼠平均新生淋巴管密度较低（*P*=0.023）。EGFR-TKI给药组小鼠新生淋巴管面积、最长径小于溶剂对照组。而EGFR-TKI对新生淋巴管的肿瘤细胞侵犯没有明显影响（*P*=0.519）。

**结论:**

EGFR-TKI可以抑制*EGFR*突变肺癌组织淋巴管的新生，抑制新生淋巴管管径及面积的扩大。

## Abstract (en)

**Background and objective:**

This study aims to explore the effect of epidermal growth factor receptor-tyrosine kinase inhibitor (EGFR-TKIs) on the lymphangiogenesis of lung cancer with *EGFR* mutation, as well as to determine the function of EGFR targeted therapy in relation to the inhibition of lymphangiogenesis during lung cancer treatment.

**Methods:**

The *EGFR* double mutant lung cancer cell line NCI-H1975 is used to construct lung cancer xenograft models. The models are divided into two groups: the solvent control group and the EGFR-TKI treatment group. Each group includes five mice. The inhibitory effect of EGFR-TKI on the growth of transplanted tumors was observed. Immunohistochemical method and lymphatic endothelium specific antibody D2-40 were used in the experiment to observe the influence of EGFR-TKI on lymphangiogenesis in lung cancer.

**Results:**

The weight and relative volume of tumors in the EGFR-TKI treated group were less than those in the solvent control group. The average lymphatic vessel density of EGFR-TKI-treated mice was 6.44 per case. This value was 10.70 per case in the solvent control group. Lower density of lymphangiogenesis was found in the EGFR-TKI treated group (*P*=0.023). The area and longest diameter of neonatal lymphatic vessel of the EGFR-TKI treated group were less than those of the solvent control group. Moreover, EGFR-TKI exhibited no significant effect on the invasion of tumor cells into the lymphatic vessel (*P*=0.519).

**Conclusion:**

EGFR-TKI can inhibit lymphangiogenesis in *EGFR* mutant lung cancer while suppressing vessel diameter and expansion area.

原发性支气管肺癌（以下简称肺癌）远处转移最常见的途径是淋巴扩散，约占50%，成为影响总体疗效的主要原因^[1]^。研究^[2]^表明，表皮生长因子受体（epidermal growth factor receptor, EGFR）高表达的肿瘤患者易出现转移，EGFR与血管内皮生长因子（vascular epidermal growth factor, VEGF）在肺癌组织中的高表达呈正相关并促进肺癌的侵袭和转移^[3]^。EGFR与VEGF表达的正相关，似乎隐约提示EGFR在肺癌新生淋巴管的生成中可能发挥了一定的作用，而EGFR酪氨酸激酶抑制剂（EGFR tyrosine kinase inhibitor, EGFR-TKI）是否具有抑制淋巴管新生和肺癌淋巴转移作用，迄今均未见报道，本文拟对此进行初步探索研究。

## 1 资料和方法

### 1.1 研究对象

细胞株及细胞培养：实验所用细胞株由中国科学院上海药物研究所培养并保存于液氮中。细胞株（NCI-H1975）培养于RPMI-1640培养液，内含10%胎牛血清、L-谷氨酰胺2 mmol/L、青霉素100 IU/mL和链霉素100 μg/mL。所有细胞均于37 ℃，5%CO_2_常规培养。实验动物：BALB/cA裸小鼠，雌性，4周-5周龄，体重（17±2）g，由中国科学院上海药物研究所提供，生产合格证编号：SCXK（沪）2008-0017；使用合格证编号：SYXK（沪）2008-0049。实验分2组，每组动物数为5只。

### 1.2 实验方法

取生长旺盛期的瘤组织剪切成1.5 mm^3^左右，在无菌条件下，接种于裸小鼠右侧腋窝皮下。裸小鼠皮下移植瘤用游标卡尺测量移植瘤直径，待肿瘤生长至100 mm^3^-200 mm^3^后将动物随机分组。BIBW2992（Gilotrif，阿法替尼）20 mg/kg组，每天口服给药1次，连续给药3周。溶剂对照组则给等量0.5%CMC-Na（羧甲基纤维素钠）。整个实验过程中，每周2次测量移植瘤直径，同时称量小鼠体重。给药3周后处死小鼠，完整取下移植瘤，石蜡包埋，准备下一步免疫组化实验。药物BIBW2992为白色粉末，每周用0.4%吐温80和0.5%羧甲基纤维素钠配置成混悬液后使用。

### 1.3 免疫组织化学实验步骤

① 石蜡切片脱蜡，水化后，用磷酸缓冲液（phosphate buffer solution, PBS）冲洗3次，每次3 min；②高压组织抗原修复（高压灭菌锅到有压力时，持续10 min，关闭高压锅，放气自然冷却）用PBS冲洗3次，每次3 min；③每张切片加50 μL 3%（20 μL PBS+80 μL甲醇+11 μL 30%过氧化氢），室温孵育10 min，用PBS冲洗3次，每次3 min；④每张切片加100 μL 10%山羊血清（900 μL PBS+100 μL山羊血清），室温孵育15 min；⑤除去10%山羊血清后，每张片子加即用型D2-40鼠抗人单克隆抗体（购自福州迈新生物公司，一抗），室温孵育60 min，PBS冲洗3次，每次3 min；⑥除去PBS后，每张片子加50 μL即用型MaxVision^TM^（羊抗鼠IgG聚合物，二抗）试剂，室温下孵育15 min，PBS冲洗3次，每次3 min；⑦除去PBS后，每张片子加100 μL新配的DAB，反应2 min，显微镜下观察；⑧自来水冲洗，苏木染色10 min，流水4 min，1%盐酸乙醇分化30 s，流水返蓝；⑨脱水，树脂封片。

### 1.4 病灶内新生淋巴管密度的定量测定

新生淋巴管密度（lymphatic vessel density, LVD）的评测根据Widner^[4]^。在显微镜下观察切片，选择目镜10倍，先在10×4倍镜下，选择有明确高表达的淋巴管区域，即“热点”的地方。每个样本依切片组织实际区域选择不低于四个热点区域，在10×20倍镜下观察，计数淋巴管数，计算平均数值即为新生淋巴管密度。肺癌灶中各区域淋巴管的密度可能是非均匀分布的，但4个“热点”地区淋巴管密度能够具有代表性。

### 1.5 新生淋巴管肿瘤侵犯的鉴定

新生淋巴管肿瘤侵犯（lymphatic vessels invasion, LVI）：如在显微镜下病灶至少有一个D2-40 IHC染色阳性的新生淋巴管管腔中存在肿瘤细胞团，即为淋巴管侵犯阳性LVI（+）^[5]^。

### 1.6 统计学方法

利用Image-Pro Plus 6.0软件，测量每个“热点”区域的新生淋巴管最长径、面积。采用SPSS 17.0统计软件进行统计，计量资料数据以Mean±SD表示，采用*t*检验。*P* < 0.05为差异有统计学意义。

## 2 结果

### 2.1 BIBW2992对人肺癌NCI-H1975荷瘤裸小鼠的肿瘤抑制作用

小鼠按照上述方法给药21天后，安乐处死，完整剥离瘤组织。图 1可见，对照组移植瘤肿瘤体积相对较大，可见肿瘤不规则生长，见分叶状。BIBW2992可以明显地抑制人肺癌NCI-H1975裸小鼠移植瘤的生长，肿瘤体积相对较小，肿瘤多呈球形，形态较规则。

**1:**
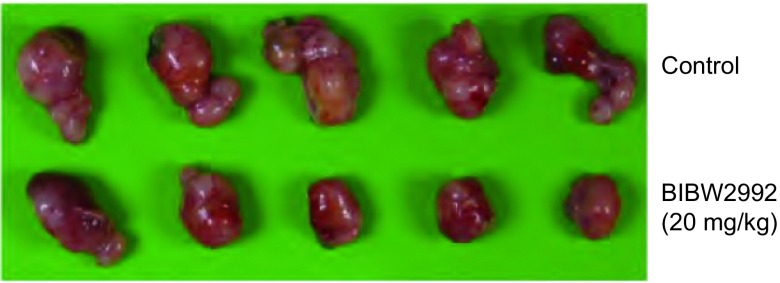
BIBW2992对于NCI-H1975裸小鼠移植瘤的生长抑制作用 Inhibitory effect of BIBW2992 on transplanted NCI-1975 in nude mice

### 2.2 BIBW2992对NCI-H1975裸小鼠移植瘤瘤量的影响

人肺癌NCI-H1975荷瘤裸小鼠给药21天之后，BIBW2992可以明显抑制小鼠肿瘤的生长，给药组小鼠瘤重为（1.37±0.55）g，而对照组的小鼠瘤重为（2.12±0.36）g，两者比较差异有统计学意义（*P*=0.035）。

### 2.3 BIBW2992对NCI-H1975裸小鼠移植瘤相对肿瘤体积的影响

图 2为两组小鼠相对肿瘤体积的对比曲线图。整个实验过程中，每周2次测量移植瘤直径。肿瘤体积（tumor volume, TV）的计算公式为：TV=1/2×a×b^2^，其中a、b分别表示长、宽。根据测量的结果计算出相对肿瘤体积（relative tumor volume, RTV），计算公式为：RTV=Vt/V0。其中V0为分笼给药时（即d0）测量所得肿瘤体积，Vt为每一次测量时的肿瘤体积。给药21天后，对照组的小鼠相对肿瘤体积为（22.48±6.85）mm^3^，给药组小鼠相对肿瘤体积为（14.75±5.61）mm^3^，给药组小鼠的相对肿瘤体积明显小于对照组，两者比较差异有统计学意义（*P*=0.031）。

**2:**
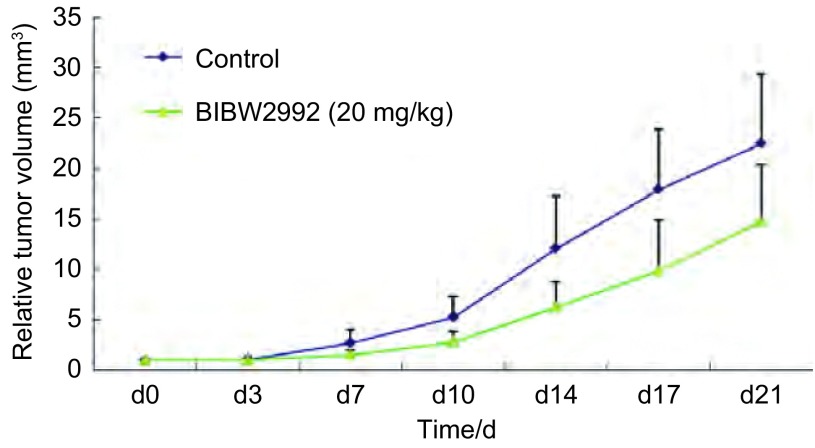
BIBW2992对人肺癌NCI-H1975裸小鼠移植瘤相对肿瘤体积的影响 Inhibitory effect of BIBW2992 on relative tumor volume in transplanted NCI-1975 in nude mice

### 2.4 NCI-H1975裸小鼠移植瘤瘤内新生淋巴管的形态学表现

在本实验10例小鼠移植瘤的瘤组织中，D2-40标记下的新生淋巴管显示为单层的、棕黄色、壁薄的管腔，管腔内没有红细胞出现。淋巴管非均匀出现在肿瘤组织中，大部分出现在肿瘤间质组织。证明D2-40标记的淋巴内皮特异性较高。裸小鼠移植瘤中新生淋巴管的密度和肿瘤细胞侵犯的形态学表现见图 3。

**3:**
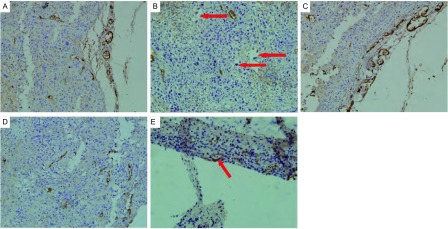
NCI-H1975裸小鼠移植瘤瘤内新生淋巴管的形态学表现。A：对照组（IHC染色，10×20）病灶内的新生淋巴管，棕黄染色，壁薄管腔，管腔内无红细胞，癌细胞团中很少出现新生淋巴管，主要位于肿瘤组织边缘；B：20 mg/kg用药组（IHC染色，10×20）病灶内新生淋巴管被棕黄染标记，而血管内皮不能被D2-40显示，血管（红色箭头所示）管壁无黄染；C：对照组（IHC染色，10×20）病灶内的新生淋巴管，管腔较大，数目较多；D：20 mg/kg给药组新生淋巴管管腔相对较小，数目相对较少（IHC染色，10×20）；E：对照组（IHC染色，10×20）瘤组织的新生淋巴管中可见肿瘤细胞侵犯（红色箭头），定义为新生淋巴管侵犯LVI（+）。 Morphological features of lymphangiogenesis in NCI-1975 transplanted nude mice. A: Control group (IHC staining, 10×20): Newborn lymphatic vessels in tumor tissue determined by immunohistochemistry, which mainly located in the tumor edge, with brown staining, thin wall, and no red blood cells in the lumen. Cancer cells seldom appear in newborn lymphatic; B: 20 mg/kg treatment group (IHC staining, 10× 20): Newborn lymphatic vessels were marked by D2-40, but vascular endothelial cannot be stained, as shown by the red arrow in the figure; C: Control group (IHC staining, 10×20): Larger and more lymphatic vessels were found in control group than those in treatment group; D: 20 mg/kg treatment group (IHC staining, 10×20): Less lymphatic vessels were found in treatment group, with smaller lumen than in the control group; E: Control group (IHC staining, 10×20): Tumor cells were found in lymphatic (red arrow), defined as the newborn lymphatic invasion LVI (+). LVI: lymphatic vessels invasion; IHC: immunohistochemistry.

### 2.5 两组NCI-H1975裸小鼠移植瘤新生淋巴管密度的比较

对照组瘤组织内新生淋巴管的密度范围为6个-21个[（10.44±3.02）个]，给药组的范围为3个-13个[（6.44±1.58）个]，两组形态特征比较见图 3C和图 3D。给药组的新生淋巴管密度明显小于对照组，差异有统计学意义（*P*=0.023）。

### 2.6 两组NCI-H1975荷瘤裸小鼠瘤组织新生淋巴管肿瘤细胞侵犯的比较

统计对照和给药两组，共10例样本，对照组病灶内见新生淋巴管肿瘤细胞侵犯2例，给药组可见1例有肿瘤细胞侵犯。进行比较发现BIBW2992对于瘤细胞的新生淋巴管侵犯的影响无统计学意义（*P*=0.519）。

### 2.7 两组NCI-H1975荷瘤裸小鼠瘤组织中新生淋巴管面积的比较

我们对不同组别的小鼠瘤组织中的新生淋巴管面积进行了比较。发现BIBW2992给药组的移植瘤瘤组织的新生淋巴管平均面积[（57.42±39.45）μm^2^]明显小于对照组[（87.29±49.72）μm^2^]，两者比较差异有统计学意义（*P*=0.006）。

### 2.8 两组NCI-H1975荷瘤裸小鼠瘤组织内新生淋巴管最长径的比较

BIBW2992给药组的移植瘤组织内新生淋巴管的最大径为（8.57±2.69）μm，明显小于对照组的新生淋巴管长径（11.13±2.98）μm，两者比较差异有统计学意义（*P*=0.004）。

## 3 讨论

肺癌经淋巴转移是最常见的转移途径，是影响临床疗效的主要原因。临床研究^[6]^表明，约80%的肿瘤有序地从原发灶通过淋巴管先蔓延淋巴结再传播到远处器官。

EGFR高表达的肿瘤患者易出现转移且复发率高^[2]^。近期研究^[3]^发现，EGFR与VEGF在非小细胞肺癌（non-small cell lung cancer, NSCLC）组织中的阳性表达呈正相关（*P* < 0.01），两者的高表达可促进肺癌的侵袭和转移，VEGF主要通过促进新生血管的形成加速肺癌的侵袭和转移。EGFR在新生淋巴管的生成中是否发挥一定的作用值得关注，研究EGFR-TKI对肿瘤组织新生淋巴管的影响，探索靶向药物抑制肺癌转移的可能性，有望为肺癌的靶向治疗提供新的研究视角。

Padera等^[7]^发现缺乏瘤内淋巴管的肿瘤仍可发生淋巴结转移，淋巴系统表面积的增大与肿瘤淋巴转移机率的增加一致，这为新生淋巴管参与肿瘤转移提供了理论依据。临床研究表明，有淋巴结转移的肺癌病灶淋巴管密度明显升高，并与较差的总体生存率相关，新生淋巴管可作为预测肺癌淋巴结转移的重要因素^[8]^，抑制肿瘤新生淋巴管能明显减少癌细胞的扩散转移^[9-11]^。本研究利用靶点突变的耐药细胞株NCI-H1975建立小鼠肺癌移植瘤模型，在抑制剂与EGFR不可逆结合，抑制肿瘤组织生长的同时，寻找其可能的新生淋巴管抑制作用。结果表明，BIBW2992给药组的移植瘤小鼠瘤内新生淋巴管密度明显少于对照组（6.44±1.58 *vs* 10.44±3.02, *P=*0.023），提示EGFR-TKI在抑制肿瘤生长的同时，明显抑制肿瘤组织内淋巴管的新生。

新生淋巴管中可见癌栓，佐证肿瘤细胞通过新生淋巴管而促进转移。本研究中给药组肿瘤细胞对新生淋巴管的侵犯和对照组相比，虽然未见统计学差异，但对照药组有3例出现肿瘤细胞侵犯新生淋巴管，而给药组仅见1例，提示EGFR-TKI对肿瘤侵犯新生淋巴管亦有一定抑制趋势，值得进一步关注。

国际肺癌分期（International Association for the Study of Lung Cancer, IASLC）（第七版）肺癌分期重点指出病灶直径在预后中的重要作用。本研究发现给药组相对肿瘤体积和瘤重均小于对照组，提示EGFR-TKI在小鼠移植瘤模型中能有效控制肿瘤的生长。同时，给药组新生淋巴管最大管径和面积均明显小于对照组，提示靶向药物可影响新生淋巴管的形态，其临床意义值得深究。非荷瘤状态的淋巴系统内，大部分毛细淋巴管处于静息塌陷状，仅有部分毛细淋巴管处于功能状态^[12]^。随着肿瘤的不断生长，瘤内局部渗透压升高，引起淋巴引流相对不足^[13]^。为了完成组织间液回流，首先使原来处于静息储备状态的毛细淋巴管大量扩张开放，并进一步促进新生毛细淋巴管的形成，以适应其对组织液运输能力的需要。同时，由于回流量大，淋巴管内压增加，使毛细淋巴管内皮细胞的连接由复杂型向简单型转变，从而有利于毛细淋巴管内皮通道的形成和对组织间物质的吸收引流^[14]^。这也为癌细胞进入新生淋巴管提供了直接进入的机会^[15]^。随着肿瘤的增大，浸润程度加重，癌内组织水肿明显，导致了瘤内新生淋巴管的管径增大、面积扩大，同时新生淋巴管密度增加，为肿瘤细胞的转移提供了有利的通道，增加了肿瘤在发展过程中淋巴道转移的机会^[14]^。有文献^[16]^报道，肿瘤生长可促进瘤内淋巴管的增生和增粗。EGFR-TKI缩减新生淋巴管管径和面积的作用对减少肺癌淋巴转移的积极意义显而易见。

EGFR-TKI阿法替尼在抑制*EGFR*突变小鼠肺癌移植瘤瘤体生长的同时，抑制瘤体内新生淋巴管生成、缩减新生淋巴管管径和面积，并有抑制肿瘤侵犯淋巴管的趋势。本研究探索了EGFR-TKI对肺癌新生淋巴管的相关影响，这是EGFR-TKI对肺癌治疗效应的崭新视角或另一重要线索，值得进一步深入研究。
